# Unveiling the dynamics and molecular landscape of a rare chronic lymphocytic leukemia subpopulation driving refractoriness: insights from single‐cell RNA sequencing

**DOI:** 10.1002/1878-0261.13663

**Published:** 2024-05-21

**Authors:** Terezia Kurucova, Kamila Reblova, Pavlina Janovska, Jakub Pawel Porc, Veronika Navrkalova, Sarka Pavlova, Jitka Malcikova, Karla Plevova, Boris Tichy, Michael Doubek, Vitezslav Bryja, Jana Kotaskova, Sarka Pospisilova

**Affiliations:** ^1^ Central European Institute of Technology, Center of Molecular Medicine Masaryk University Brno Czech Republic; ^2^ Department of Experimental Biology, Faculty of Science Masaryk University Brno Czech Republic; ^3^ Department of Internal Medicine, Hematology and Oncology, Faculty of Medicine Masaryk University and University Hospital Brno Czech Republic; ^4^ Institute of Medical Genetics and Genomics, Faculty of Medicine Masaryk University and University Hospital Brno Czech Republic

**Keywords:** CLL, clonal evolution, rare subpopulation, refractoriness, single‐cell RNA sequencing

## Abstract

Early identification of resistant cancer cells is currently a major challenge, as their expansion leads to refractoriness. To capture the dynamics of these cells, we made a comprehensive analysis of disease progression and treatment response in a chronic lymphocytic leukemia (CLL) patient using a combination of single‐cell and bulk genomic methods. At diagnosis, the patient presented with unfavorable genetic markers, including notch receptor 1 (*NOTCH1*) mutation and loss(11q). The initial and subsequent treatment lines did not lead to a durable response and the patient developed refractory disease. Refractory CLL cells featured substantial dysregulation in B‐cell phenotypic markers such as human leukocyte antigen (HLA) genes, immunoglobulin (IG) genes, CD19 molecule (*CD19*), membrane spanning 4‐domains A1 (*MS4A1*; previously known as CD20), CD79a molecule (*CD79A*) and paired box 5 (*PAX5*), indicating B‐cell de‐differentiation and disease transformation. We described the clonal evolution and characterized in detail two cell populations that emerged during the refractory disease phase, differing in the presence of high genomic complexity. In addition, we successfully tracked the cells with high genomic complexity back to the time before treatment, where they formed a rare subpopulation. We have confirmed that single‐cell RNA sequencing enables the characterization of refractory cells and the monitoring of their development over time.

## Graphical abstract

We studied a patient with chronic lymphocytic leukemia by using a combination of single‐cell and bulk genomic methods. We analyzed B‐cells and DNA from time of diagnosis (DG), progression (PR), relapse (RL) and refractory disease phase (RF), and DNA from second relapse (RL2). We reconstructed the clonal evolution and tracked the cells with high genomic complexity (HGC) back to the pre‐treatment time.

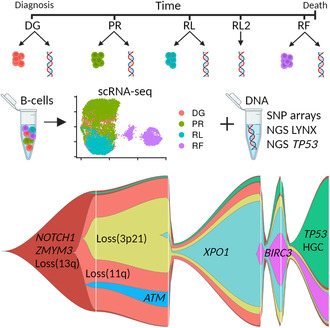

**Abbreviations**
ALZ: alemtuzumab
BR: bendamustine‐rituximab
CLL: chronic lymphocytic leukemia
CNA: copy number aberration
COP: cyclophosphamide‐vincristine‐prednisone
DG: diagnosis
DGE: differential gene expression
DGV: database of genomic variants
FASAY: functional analysis of separated alleles in yeast
FCR: fludarabine‐cyclophosphamide‐rituximab
FISH: fluorescence *in situ* hybridization
IGHV: immunoglobulin heavy chain variable
LOH: loss of heterozygosity
NGS: next‐generation sequencing
OS: overall survival
PR: progression
RF: refractory disease phase
RL: first relapse
RL2: second relapse
scRNA‐seq: single‐cell RNA sequencing
TP: time points
VAF: variant allele frequency

## 1 Introduction

Chronic lymphocytic leukemia (CLL) is a lymphoproliferative disease primarily affecting the elderly population in Western countries. It is characterized by the accumulation of small, morphologically mature, yet non‐functional B‐lymphocytes, in peripheral blood, bone marrow, lymph nodes, and spleen. CLL clinical manifestations vary greatly, with some patients remaining asymptomatic and under observation for several years, while others experience an aggressive disease course, necessitating early therapy intervention. Still, CLL remains an incurable condition, and the overall survival (OS) rate ranges from a few months to several years.

The highly variable clinical behavior of CLL can be attributed to its molecular heterogeneity. Roughly 80% of patients exhibit one or more recurrent genomic abnormalities, including del(13q), del(11q), del(17p), and +12 [1]. Additionally, CLL patients can be categorized into two groups based on the presence or absence of somatic hypermutation in the immunoglobulin heavy chain variable (IGHV) genes, with unmutated IGHV being linked to faster disease progression and shorter OS [2, 3]. Advancements in high‐throughput sequencing have unveiled several frequently mutated genes in CLL, such as *SF3B1*, *TP53*, *NOTCH1*, *XPO1*, *BIRC3*, *ATM*, *MYD88*, *POT1*, *NFKBIE* and *ZMYM3* [4, 5, 6]. Adverse prognostic implications have primarily been observed for *TP53*, *ATM*, *SF3B1*, *NOTCH1*, and *BIRC3* mutations [7]. However, current treatment guidelines for CLL patients primarily consider the presence of recurrent chromosomal abnormalities, the IGHV mutational status, and *TP53* gene mutations when making therapeutic decisions [8, 9].

As with other malignancies, the genome of CLL is dynamic, and the genetic abnormalities, together with their prevalence, may evolve throughout the disease course. This clonal evolution often leads to the emergence of more aggressive clones or subclones. Clonal diversity plays a significant role in cancer progression, particularly when the tumor cells are exposed to selective pressures such as chemotherapy, favoring the selection and expansion of a resistant clone [10, 11]. Detecting the presence of such resistant cells, which could have a significant impact on cancer progression, at the time of diagnosis is very challenging because conventional genomic techniques such as genomic/chromosomal microarrays and bulk next‐generation sequencing (NGS) average the signal from several cell populations. In contrast, single‐cell RNA sequencing (scRNA‐seq) offers individual cell resolution, which presents immense potential in identifying these rare cells and facilitating further investigation.

In this study, we aimed to investigate the possibility of utilizing a combination of diverse high‐throughput sensitive methods, primarily scRNA‐seq, for unveiling the dynamics of CLL disease. We have shown that employing these methods makes it possible to reconstruct clonal evolution, characterize the resistant cells and to track them back in time, therefore may help to elucidate the mechanism of treatment resistance and lead to more personalized therapy approach.

## 2 Materials and methods

### 2.1 CLL samples processing

**Fig. 1:**
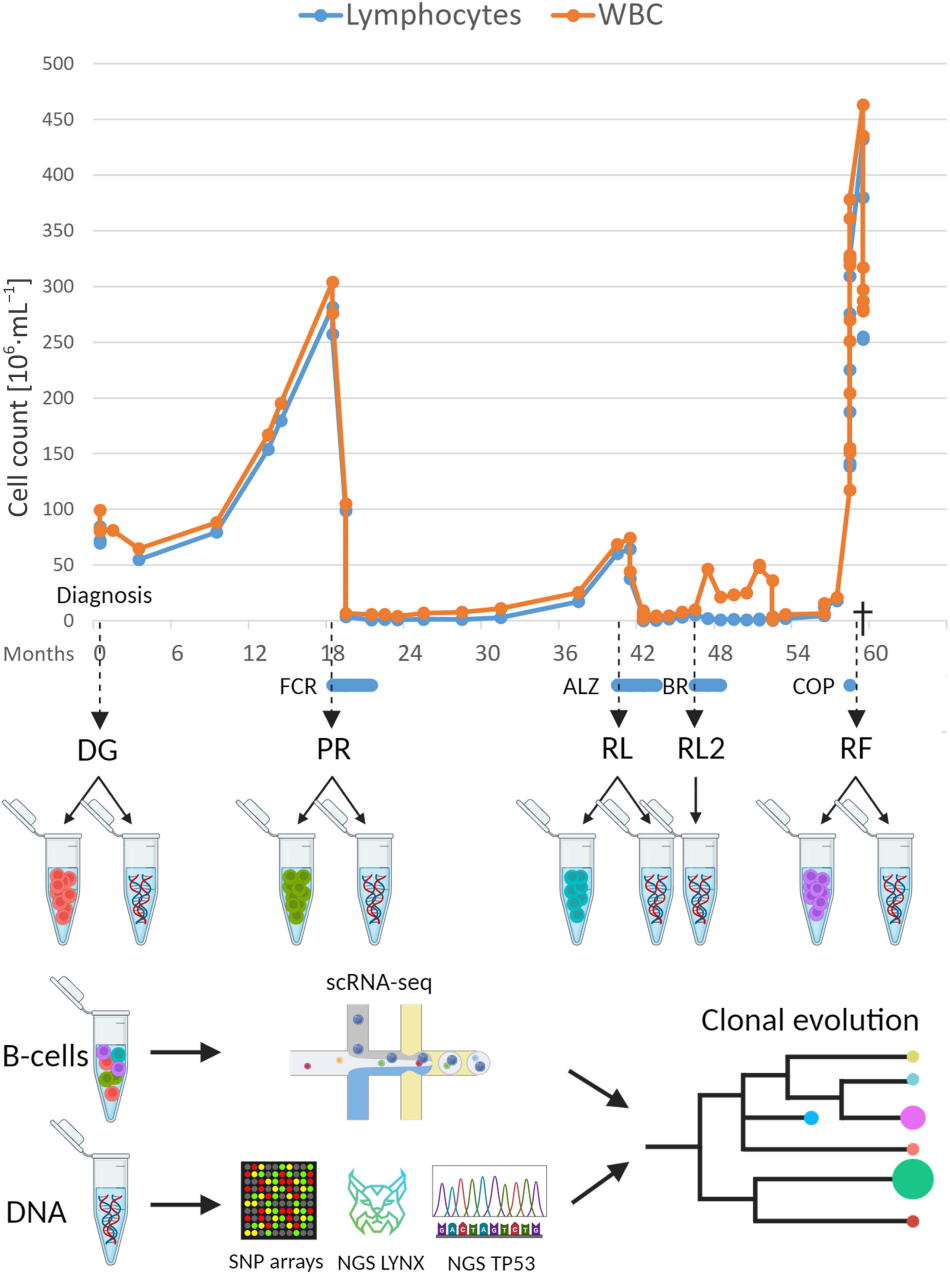
Disease evolution and case study design. The graph captures a fluctuation of white blood cell and lymphocyte counts during the disease course, with different therapy periods and the individual time points included in the study indicated along the timeline. Below the time points is shown which material was processed from the given sample (B‐cells or DNA) and which analyzes were performed on the given material (single‐cell RNA sequencing/SNP arrays and NGS sequencing of the LYNX panel and *TP53* amplicons). The combination of the results finally enabled the reconstruction of the disease clonal evolution in the patient. ALZ, alemtuzumab; BR, bendamustine‐rituximab; COP, cyclophosphamide‐vincristine‐prednisone; DG, diagnosis; FCR, fludarabine‐cyclophosphamide‐rituximab; NGS, next‐generation sequencing; PR, progression; RF, refractory disease phase; RL2, second relapse; RL, first relapse; scRNA‐seq, single‐cell RNA sequencing; SNParray, single nucleotide polymorphism array; WBC, white blood cell count.

A retrospective case study subject was diagnosed at the Department of Internal Medicine, Hematology and Oncology, University Hospital Brno. The patient was monitored and treated according to the valid iwCLL guidelines [12]. A written informed consent for storing and use of the processed biological material for scientific purposes was obtained. The consent and study methodologies conformed to the standards set by the Declaration of Helsinki and were approved by the Ethical Committee of the University Hospital Brno (05‐240 619/EK). Relevant clinical information related to the disease course and treatment was collected. Samples were collected from April 2012 to March 2017. B‐cells were separated from peripheral blood using the RosetteSep™ Human B Cell Enrichment Cocktail (Stemcell Technologies, Vancouver, Canada) and Ficoll‐Paque PLUS (GE Healthcare, Chicago, IL, USA) density gradient centrifugation. The resulting purity of B‐cells was assessed as > 95% by flow cytometry. Finally, 5 million cells were used for DNA isolation (MagCore Genomic DNA Whole Blood Kit, RBC Bioscience, Taipei, Taiwan) and the remaining cells were cryopreserved. B‐cells and DNA were stored at five consecutive time points (TPs): from the time of diagnosis (DG), during progression shortly before the frontline therapy with FCR (fludarabine‐cyclophosphamide‐rituximab) (PR), in relapse before initiating therapy with ALZ (alemtuzumab) (RL), in second relapse before initiating therapy with BR (bendamustine‐rituximab) (RL2), and at the time of refractoriness after several therapy lines (RF) (Fig. 1). RL2 was only used for bulk analyses (i.e. genomic microarrays, targeted panel sequencing and *TP53* sequencing), as cells were no longer available at the time the study was performed.

### 2.2 scRNA‐seq library preparation and sequencing

Frozen cells were thawed and prepared for scRNA‐seq. Cell concentration and viability were estimated using a Bürker chamber with the addition of TrypanBlue. If viability was < 80%, EasySep™, Dead Cell Removal (Annexin V) Kit (Stemcell Technologies) was used to increase the proportion of viable cells. Next, the cells from individual TPs were stained with different TotalSeq‐B Hashtag antibodies (B0251‐B0260; BioLegend, San Diego, CA, USA) according to the manufacturer's protocol, and proportionally pooled, allowing multiplexing of cells from different TPs into a single experiment. Subsequently, a scRNA‐seq library was generated using the Chromium Controller (10x Genomics, Pleasanton, CA, USA), and Next GEM Single Cell 3′ Library & Gel Bead Kit v3.1 (10x Genomics) following the manufacturer's instructions and targeting approximately 10 000 cells per experiment. In total, cells from four consecutive TPs (DG, PR, RL, and RF) were processed in three experiments. The library quantity and size distribution were checked using QuantiFluor dsDNA System (Promega, Madison, WI, USA) and Fragment Analyzer DNF‐474 High Sensitivity NGS Fragment Analysis Kit (Agilent Technologies, Santa Clara, CA, USA). Libraries were sequenced on NextSeq 500 using High Output Kit v2.5 (75 cycles, Illumina, San Diego, CA, USA) with an average coverage of 30 000 reads per cells.

### 2.3 scRNA‐seq data processing and analysis

Fastq files were processed and aligned to human reference refdata‐gex‐GRCh38‐2020‐A (10x Genomics) using cell ranger v5.0.1 software (10x Genomics). seurat package v4.0.5 [13] was used in the subsequent step for data cleaning and processing, visualization, and calculation of differential gene expression (DGE). First, cells were demultiplexed based on TotalSeq‐B hashtag sequences, while doublets (cells containing sequences of two different hashtags) and negative cells were removed from the analysis. Furthermore, cells with less than 200 expressed genes and more than 25% mitochondrial reads were discarded. After these quality control steps, cells from the four consecutive TPs sequenced in different experiments were merged into a single analysis. In the next steps, the counts were normalized, scaled, and entered into a PCA analysis calculated on the basis of the 2000 most variable genes. The top 20 principal components were used for UMAP visualization and nearest‐neighbor graph construction. If a separate cluster containing cells with a high proportion of mitochondrial RNA was formed, such cluster was removed, and the data object was recalculated. DGE was calculated in Seurat using the MAST test [14]. The estimation of copy number aberration from scRNA‐seq data was performed using the infercnv package v1.19.1 (inferCNV of the Trinity CTAT Project; https://github.com/broadinstitute/infercnv).

Since in our dataset, three experiments containing cells from four time points were combined in one analysis, the presence of batch effect was evaluated. As cells from the PR TP were processed twice in two separate experiments, these cells were used to visualize the batch effect (Fig. S1). Two batches of PR cells from the same TP entirely overlapped when visualized in the Fig. S1, therefore the batch effect was considered negligible.

### 2.4 Genomic microarrays analysis

DNA from each TP was used for genome‐wide analysis of copy number aberrations (CNA) and losses of heterozygosity (LOH) using the genomic microarray CytoScan HD and the respective reagent bundle (Thermo Fisher Scientific, Waltham, MA, USA) according to the manufacturer's protocol. During the sample processing, the following modifications were implemented: DNA input was increased to 300 ng, and the Titanium DNA Amplification Kit (Clontech, Mountain View, CA, USA) was used instead of the CytoScan Amplification Kit for DNA amplification after adaptor ligation. The hybridization, washing and staining, and scanning were performed using the GeneChip Instrument System. Data were analyzed in chromosome analysis suite software (version 4.3) with the NetAffx Genomic Annotation hg19 (version 20 220 623). Genomic abnormalities were inspected while filtering in CNAs > 20 kbp and LOHs > 5 Mbp in a default setting and were further compared against the Database of Genomic Variants (DGV) and ChASDB aDGV healthy individuals' dataset. The final sets of presumably somatic, CLL‐associated genomic abnormalities were recorded according to the standard ISCN 2020 nomenclature.

### 2.5 Integrative LYNX panel targeted sequencing

High‐throughput sequencing of target regions relevant to lymphoid malignancies was carried out using a capture‐based NGS panel lynx [15]. In short, the sequencing library was prepared using SureSelect XT HS kit (Agilent Technologies) with a custom probe set enabling detection of genome‐wide chromosomal aberrations, recurrent aberrations including del(17p), del(11q), del(13q) and +12, the assessment of immunoglobulin gene rearrangements, and the mutational analysis of 67 protein‐coding genes. LYNX libraries were sequenced on NextSeq 500 using Mid Output Kit v2.5 (300 cycles; Illumina), and subsequent data analysis was done using a dedicated in‐house bioinformatic pipeline [15]. A plot capturing estimated clonal evolution was created using the fish plot package [16].

### 2.6 *TP53* amplicon sequencing

Isolated DNA from each TP was used for deep NGS sequencing of *TP53* gene, as described previously [17, 18, 19]. In brief, eight amplicons covering exons 2–11 were pooled and subsequently used as an input for library preparation with the Nextera XT DNA Sample Preparation Kit (Illumina). *TP53* amplicon libraries were sequenced on the MiSeq instrument using MiSeq Reagent Kit v2 (300 cycles; Illumina). Variants were called using an established in‐house bioinformatic pipeline [19].

## 3 Results

### 3.1 Case description

A 46‐year‐old female presented to the hematological clinic with leukocytosis (80.2 × 10^6^·μL^−1^), splenomegaly, and lymphadenopathy in April 2012. The patient was diagnosed with CLL, stage Rai II. Fluorescence *in situ* hybridization (FISH) analysis revealed the presence of del(11q) and del(13q) in 65% and 91% of cells, respectively. The *TP53* mutational status was assessed as wild‐type by functional analysis of separated alleles in yeast (FASAY) used as standard diagnostic method [19, 20]. The clonotypic IGH rearrangement with the unmutated IGHV somatic hypermutation status (100% identity to germline) was reported. Subsequently, disease progression was observed and a frontline treatment with three cycles of FCR was initiated 18 months after diagnosis. Although a complete response was achieved, the patient relapsed after 22 months and subsequently received second‐line treatment with ALZ. Despite this treatment, CLL progression was observed again. The patient did not agree with an allogeneic transplant, thus was scheduled for treatment with three cycles of BR, which resulted in partial remission. One year later, complex chromosomal changes in CLL cells were reported in disease relapse, including newly acquired del(17p) in 43% of cells. The non‐functionality of *TP53* was confirmed by FASAY. While Ibrutinib treatment was being approved, the patient was treated with dexamethasone and COP (cyclophosphamide‐vincristine‐prednisone) for persistent lymphadenopathy and thrombocytopenia. Progression to acute lymphoblastic leukemia (due to the presence of 3% blasts in the peripheral blood) was suspected but not confirmed by bone marrow flow cytometry analysis. Transformation to Richter's syndrome was not tested. Ultimately, the patient passed away 5 years from diagnosis in 2017, following fulminant CLL progression.

### 3.2 ScRNA‐seq revealed a striking change in clonal dominance during the refractory disease phase

**Fig. 2:**
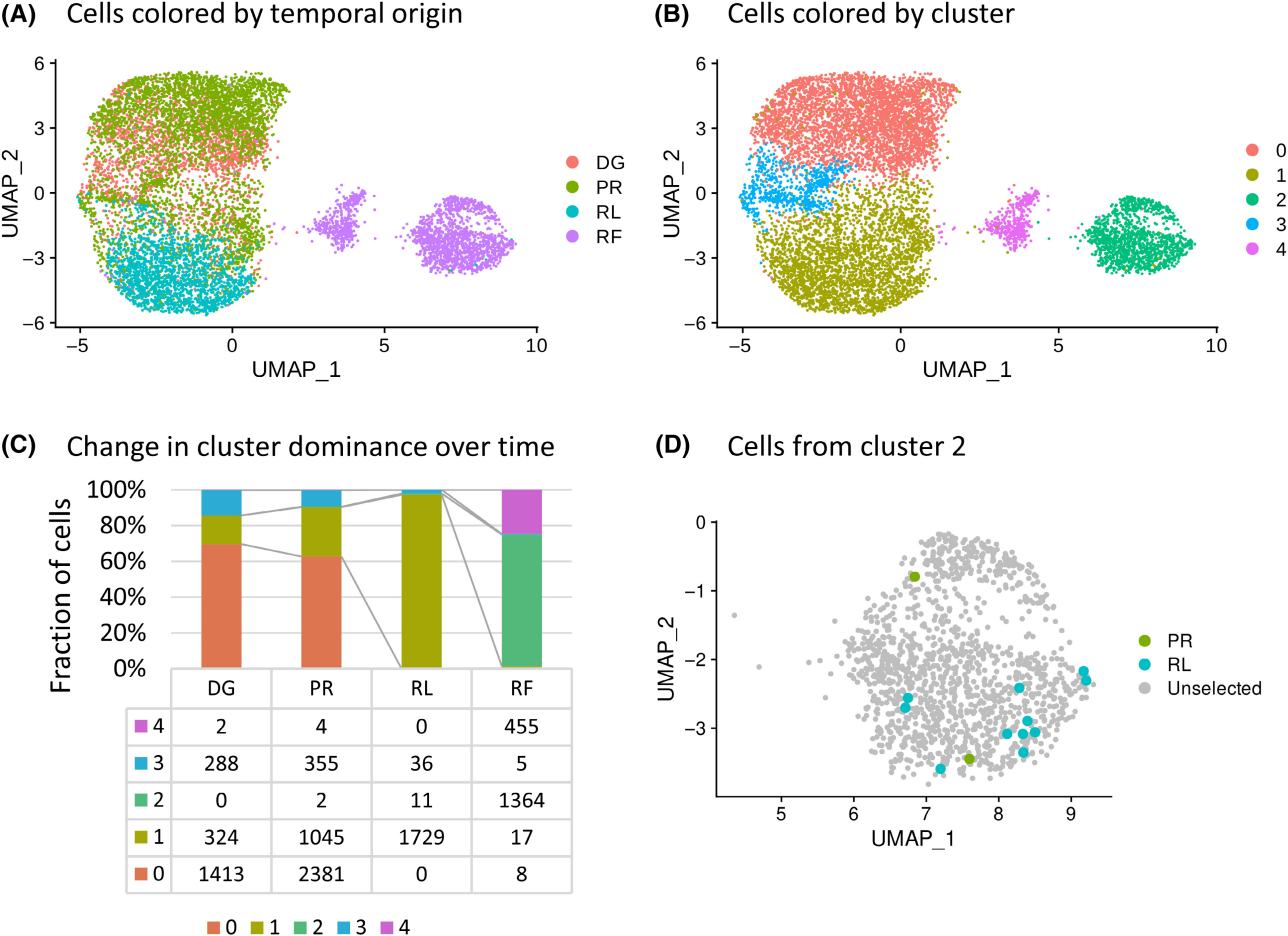
Visualization and review of single‐cell RNA sequencing data. (A) Final UMAP visualization of the single‐cell RNA sequencing data with cells colored according to their temporal origin. (B) UMAP visualization of scRNA‐seq data with cells color‐coded into 5 clusters according to their expression similarity. (C) Bar plot capturing the proportion of cells from each expression cluster in individual time points and the change in cluster dominance over time with table of total cell counts of individual time points in expression clusters. (D) Cells from expression cluster 2 with PR and RL cells presence highlighted. DG, diagnosis; PR, progression; RF, refractory disease phase; RL, first relapse.

B‐cells from four consecutive TPs were analyzed by scRNA‐seq (Fig. 1). DG and PR cells overlapped in the 2D UMAP plot, RL cells overlapped only partially with the previous TPs, while RF cells were clearly separated (Fig. 2A). Based on gene expression, the cells were divided into five clusters as shown in Fig. 2B. The proportion of cells from each TP in the individual clusters and the change in cluster dominance over time are depicted in Fig. 2C. Cells from cluster 0 dominated in the DG and PR, accounting for an average of 62% of cells, while cells from cluster 1 expanded over time to comprise more than 90% of cells in the RL point (Fig. 2C). At the RF, two new clusters 2 and 4 emerged, together containing 92% of the cells.

### 3.3 DGE analysis highlighted the upregulation of apoptosis inhibitors and the downregulation of B‐cell phenotypic markers in the refractory phase

**Fig. 3:**
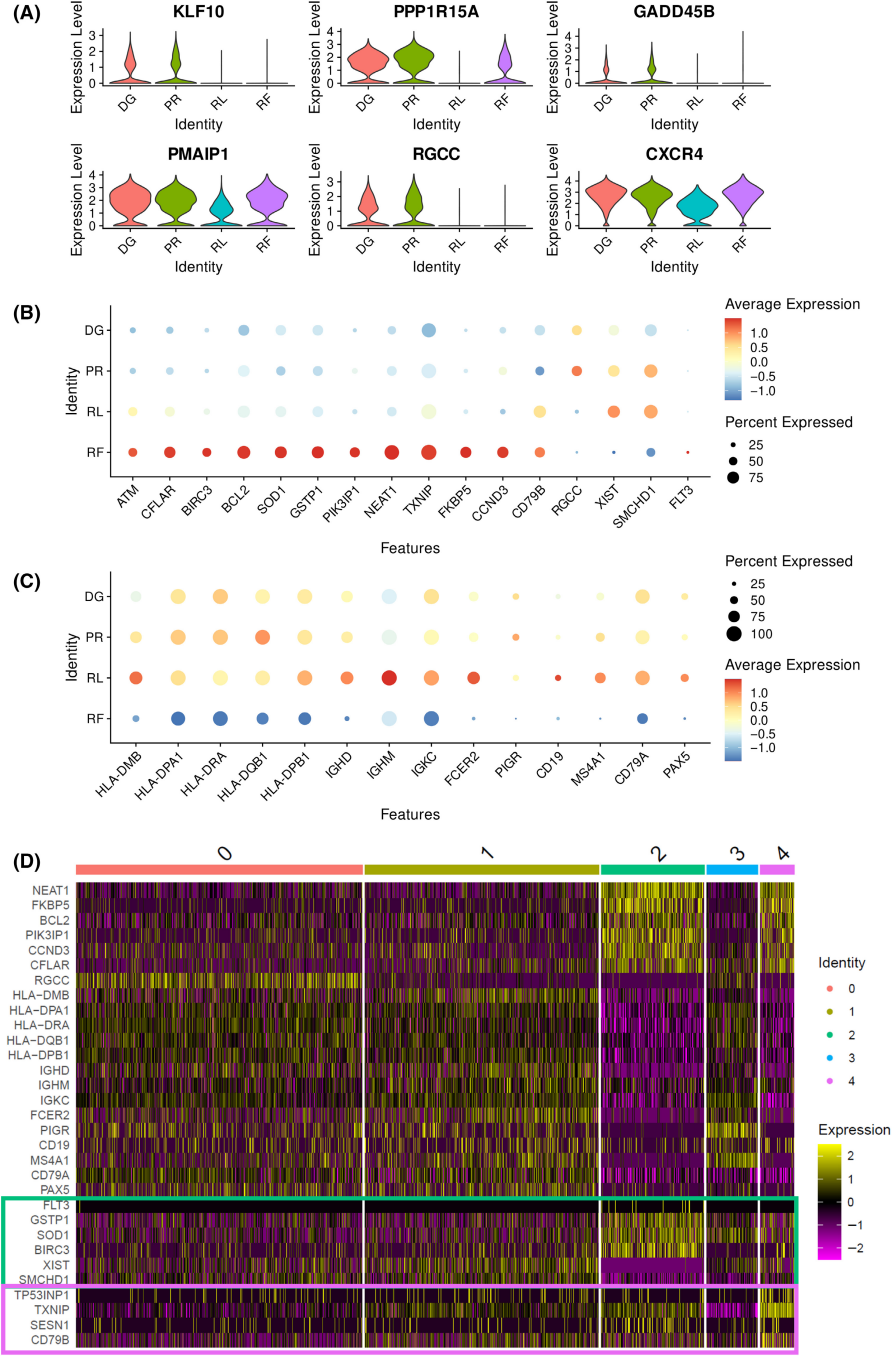
Differential gene expression analysis based on single‐cell RNA sequencing. Cells from different time points were subjected to differential gene expression analysis. (A) Violin plots show genes significantly altered when comparing RL cells with DG and PR cells (using MAST test with absolute average log2FC > 0.9 and *P* value adjusted < 0.001). (B) Dot plot of genes significantly changed in RF cells compared to other cells (using MAST test with *P* value adjusted < 0.001). (C) Dot plot of genes altered in RF cells related to B‐cell phenotype. (D) Heatmap of genes changed in RF cells with emphasis on the difference between cluster 2 and 4. Genes differentially expressed in cluster 2 are marked in a green box and in cluster 4 in a purple box. DG, diagnosis; PR, progression; RF, refractory disease phase; RL, first relapse.

DGE analysis was performed comparing cells from different TPs. No significant differences were observed between DG and PR cells. RL cells showed downregulation of several tumor suppressor genes (Fig. 3A) including *KLF10*, *PPP1R15A*, and *GADD45B*. When comparing the RF cells to DG and PR cells, genes associated with inhibition of apoptosis (*CFLAR*, *BIRC3*, and *BCL2*), detoxification (*SOD1*, *GSTP1*), and other processes were found to be upregulated. In addition, there was a notable downregulation of several B‐cell markers (Fig. 3C), indicating a loss of the original B‐cell phenotype in RF cells. RNA expressions of significantly changed genes of interest at RF are shown in Fig. 3B,C. Differentially expressed genes with values of log2 fold change are listed in Tables S1 and S2, showing RF vs. remaining TPs, and RL vs. DG + PR, respectively.

Since the majority of RF cells were divided into two separate clusters (cluster 2 and cluster 4), the similarity and difference of expression between these clusters were further investigated. Both clusters showed downregulation of B‐cell markers, while genes encoding detoxification enzymes were upregulated in cluster 2 and tumor suppressor genes were upregulated in cluster 4 (Fig. 3D).

### 3.4 Detecting the high genomic complexity in the fraction of RF cells and tracking their presence over time through CNA estimation

**Fig. 4:**
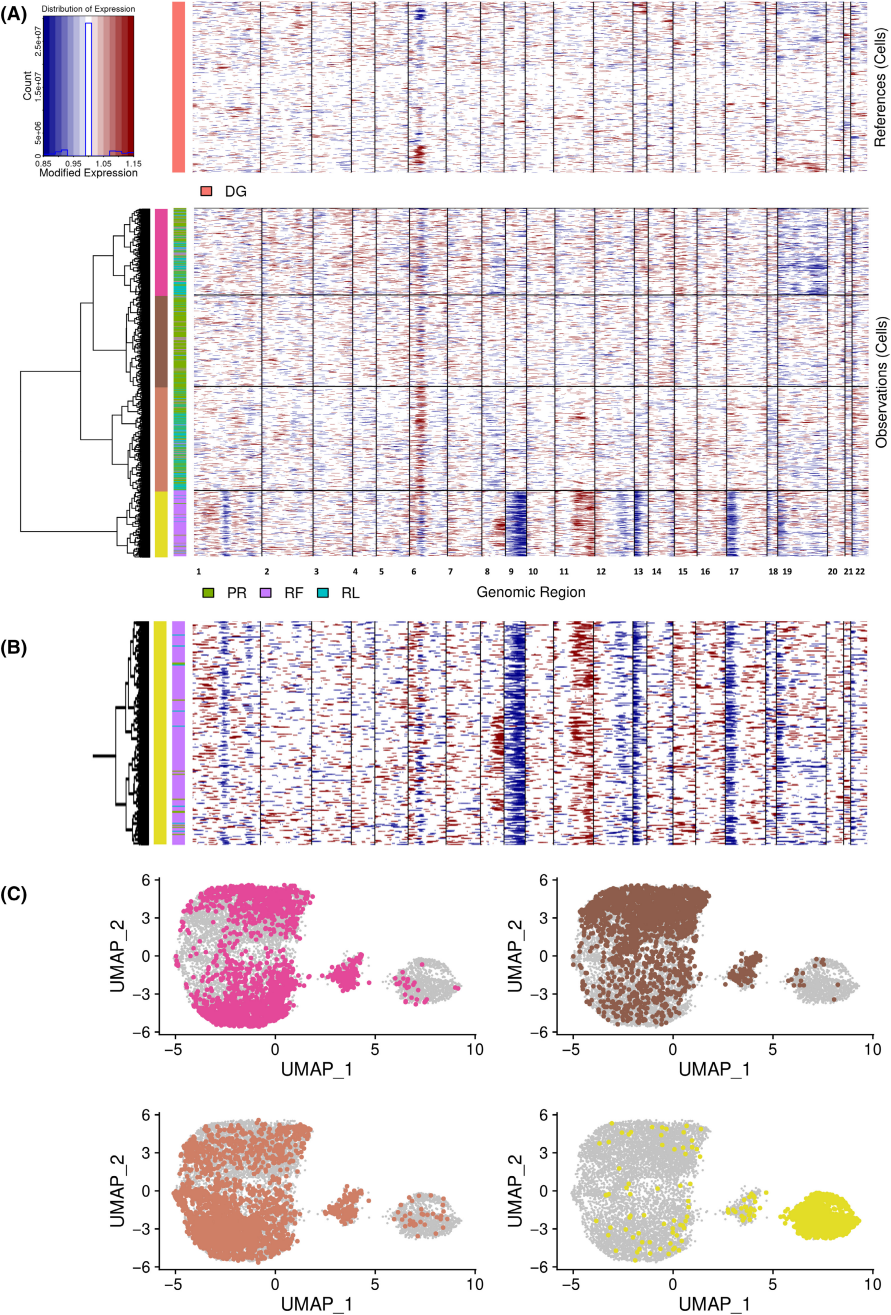
Graphical representation of CNA estimation from single‐cell RNA sequencing data by the InferCNV tool using unsupervised clustering. (A) Cells divided into time points were subjected to unsupervised clustering based on estimation of copy number aberration (CNA). Inside the heat maps, the cells are located on the *y*‐axis and ordered chromosomes on the *x*‐axis. The upper heat map shows DG cells that were used as a reference and the lower heat map shows cells from follow‐up time points (PR, RL, and RF) divided into new four color‐coded InferCNV clusters based on a similar CNA profile: cyclamen, brown, orange, and yellow. (B) Stretched yellow InferCNV cluster harboring high genomic complexity showing the presence of PR and RL cells within this cluster. (C) Cells from the newly created four InferCNV clusters visualized in the original UMAP with the corresponding color annotation preserved. DG, diagnosis; PR, progression; RF, refractory disease phase; RL, first relapse.

The next step involved performing CNA estimation using the InferCNV tool, with DG cells used as the reference. Interestingly, CNA estimation revealed presence of high genomic complexity in cells from cluster 2 (Fig. S2). As indicated by clustering based on gene expression, 11 RL cells and two PR cells (highlighted in Fig. 2D) were assigned to the aberrant cluster 2, prompting further investigation into the indication of the high genomic complexity cells in other TPs. Therefore, InferCNV with unsupervised hierarchical clustering was performed, using DG cells as a reference. Consequently, 1.2% of PR cells (44 cells) and 1.9% of RL cells (34 cells) were assigned to the aberrant cluster with high genomic complexity profile, along with 76% of RF cells (1400 cells) (Fig. 4A,B). Figure 4C highlights cells divided into four new color‐coded InferCNV clusters generated by unsupervised clustering in original UMAP. A comparison of cell barcodes revealed that two PR cell and seven RL cells assigned to expression cluster 2 matched cells in the InferCNV cluster with high genomic complexity.

### 3.5 Genomic microarrays verified CNA estimation from scRNA‐seq data and confirmed the presence of rare population of cell during pre‐treatment progression

**Fig. 5:**
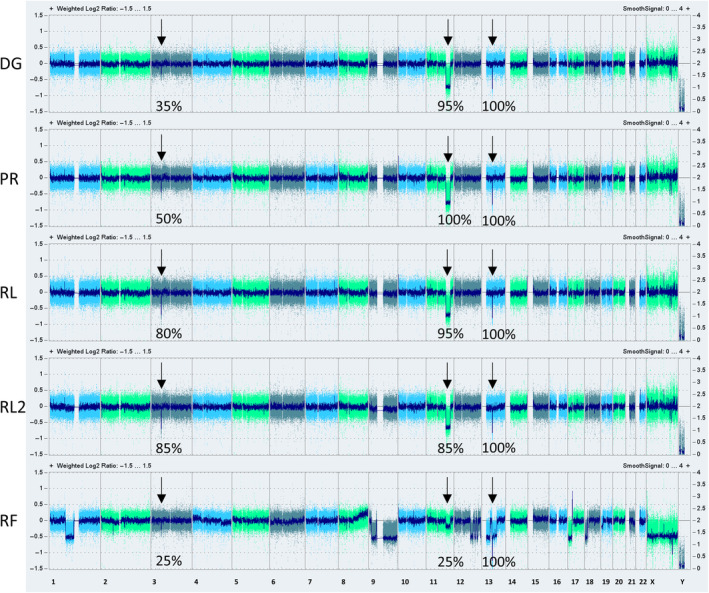
Graphical representation of CNA detection by genomic microarrays. Visualization of copy number aberration (CNA) results from genomic arrays for individual samples of B‐cells, with ordered chromosomes located on the *x*‐axis. Aberrations that were present from the beginning of the disease (loss(3p21), loss(11q) and loss(13q)) are marked by arrows and their frequency in percentage. DG, diagnosis; PR, progression; RF, refractory disease phase; RL, first relapse; RL2, second relapse.

To validate the CNA estimation obtained from scRNA‐seq, highly sensitive CytoScan HD arrays were employed. The arrays confirmed multiple genomic changes at RF, indicating genomic instability. Furthermore, the arrays revealed the persistence of losses in 3p21, 11q, and 13q throughout the whole disease course. High genomic complexity with multiple alterations such as loss(1p), loss(9p), loss(17p), monosomy X, and chromothripsis in 12q and 18p was detected in the RF TP. The frequencies of individual aberrations at different TPs are depicted in Fig. 5 and summarized in Table S3. Interestingly, while loss(11q) was present in approximately 95% and 100% of cells at diagnosis and progression, its proportion dropped to 25% at refractory disease stage, confirming the presence of rare subpopulation without loss(11q) in pre‐treatment time. At the same time, the proportions of detected aberrations and their changes over time corresponded to the results from InferCNV and point to the presence of two populations at the time of the refractory disease phase: one with loss(11q) and the other without loss(11q) containing the high genomic complexity (as resulted from InferCNV).

To determine the specific time or therapy that triggered the expansion of the non‐loss(11q) population, an additional TP RL2 was included in the bulk analyses, even though cells from this TP were not available for scRNA‐seq. According to genomic array data, the proportion of non‐loss(11q) population increased slightly after ALZ treatment (Table S3), but their expansion only occurred after BR treatment at RF.

Visual comparison of genomic microarrays CNA results with scRNA‐seq CNA estimation showed that InferCNV did not detect loss(chrX) and poorly delineated loss(12q) and loss(18p). Discrepancy in results occurred in the 11q region, with inferCNV showing a gain of this region, while genomic arrays showed the opposite. ScRNA‐seq estimation, using DG cells as a reference, showed a higher signal of the 11q region in RF cells due to a decrease in the number of cells with loss(11q). In this regard, the output from InferCNV was correct. To sum up, the CNA estimation from scRNA‐seq via InferCNV proved to be a reliable tool for CNA detection. The disadvantage of this approach was lower sensitivity and insufficient resolution to denote region affected by genomic abnormality, as the presence of aberration was estimated only from transcribed regions of the genome. In addition, the signals on some chromosomes were noisy (e.g. chr6 and chr19) and prevented CNA assignment.

### 3.6 LYNX panel revealed mutations in several genes including *ATM* , *NOTCH1* , *ZMYM3* , *TP53* , and *BIRC3*

To gain insight into the mutational landscape of individual TPs, the lymphoid NGS panel LYNX was employed. The presence of mutations in the *NOTCH1* and *ZMYM3* genes, with a variant allele frequency (VAF) of 50% detected at DG, was revealed. The frequencies of these mutations remained stable over time and increased only at RF, reaching 80% due to acquired loss(9q) and loss(Xq), where the genes are located. Furthermore, during the course of the disease, the mutation in *ATM* disappeared and new mutations appeared in other genes, namely *XPO1*, *TP53*, and *BIRC3* (Table 1). Clonality of the IG rearrangement stayed the same during the whole disease course (Table 1).

**Table 1:** Summary of detected variants with their allelic frequencies at individual time points obtained by sequencing the LYNX panel and *TP53* amplicons. DG, diagnosis; IGH, immunoglobulin heavy locus; IG, immunoglobulin gene; IGK, immunoglobulin kappa locus; PR, progression; RF, refractory disease phase; RL2, second relapse; RL, first relapse; VAF, variant allele frequency.

LYNX					VAF (%)				
Gene	Cytogenetic location	cDNA change	Protein change	Transcript ID	DG	PR	RL	RL2	RF
*NOTCH1*	9q34.3	c.7541_7542del	p.Pro2514ArgfsTer4	ENST00000651671	50	50	50	50	80
*ZMYM3*	Xq13.1	c.2626C > T	p.Pro876Ser	ENST00000314425	50	50	50	50	80
*ATM*	11q22.3	c.3077 + 1G > T	p.?	ENST00000278616	5	10			
*XPO1*	2p15	c.1711G > A	p.Glu571Lys	ENST00000401558		2	35	35	10
*TP53*	17p13.1	c.582del	p.Ile195SerfsTer52	ENST00000269305			2	5	60
*BIRC3*	11q22.2	c.1664G > T	p.Arg555Ile	ENST00000263464			15	30	10
*RB1*	13q14.2	c.1735C > T	p.Arg579Ter	ENST00000267163					8

*TP53* amplicon sequencing					VAF (%)				
Gene	Cytogenetic location	cDNA change	Protein change	Transcript ID	DG	PR	RL	RL2	RF
*TP53*	17p13.1	c.582del	p.Ile195SerfsTer52	ENST00000269305	0.9	0.2	3.0	7.6	64

IG rearrangement analysis					Clone proportion (%)				
Locus	V gene	D gene	J gene	Junction	DG	PR	RL	RL2	RF
IGH	IGHV5‐10‐1	IGHD6‐19	IGHJ4	CARHQPLGIAEPLDYW	93	96	99	93	93
IGK	IGKV1‐39	–	IGKJ1	CQQSYSTPPKTF	95	95	100	94	95

Since the *TP53* mutational profile of this patient was retrospectively reanalyzed in our previous study [11], we used this opportunity and obtained the data from ultrasensitive *TP53* sequencing for more sensitive detection of this mutation during the disease course. Results revealed the subclonal presence of the same mutation detected during the refractory period at DG, with a VAF of 0.9%. The change in the frequency of this variant over time is documented in Table 1. The frequencies are consistent with findings from scRNA‐seq and genomic arrays and point to striking change in cell populations present in the refractory phase of the disease.

## 4 Discussion

In this study, we conducted a comprehensive analysis of a CLL patient's disease evolution by combining single‐cell and bulk genomic methods. The complexity of the presented case highlights the need for a combination of different sensitive methods to examine in detail the effect of treatment on clonal evolution in CLL patients, particularly those with aggressive forms of the disease.

The disease of the presented patient case exhibited several unfavorable markers at DG, including the presence of *NOTCH1* mutation in all malignant cells and loss(11q) in 95% of cells, as revealed by genomic microarrays. Throughout the disease course, the patient underwent several therapies that did not have a long‐term effect and resulted in refractory disease. In terms of gene expression, cells from this refractory phase were completely different from cells from previous TPs. Striking downregulation in the expression of B‐cell markers collectively suggests alterations in B‐cell phenotype and function at RF. Downregulation of *PAX5* and upregulation of *FLT3* suggests de‐differentiation state of RF cells and transformation of the disease, as *Pax5* deficiency was proved to lead to de‐differentiation of B‐cell and formation of aggressive lymphomas in mice [21]. Further studies are needed to elucidate the underlying mechanisms driving these changes in gene expression and their functional implications in refractory CLL.

In addition to expression changes, around a quarter of RF cells showed high genomic complexity. Remarkably, clustering based on both expression and CNA estimation assigned few cells from the PR and RL to the cluster with high genomic complexity. Based on this finding, we inferred that high genomic complexity cells were present as a rare subpopulation already before therapeutic intervention, while the selection pressure of several therapy lines enabled their expansion. This conclusion was subsequently supported by the genomic array, as a population of cells without loss(11q) dominated at RF, although this loss was present with frequency of 95–100% at DG and PR.

**Fig. 6:**
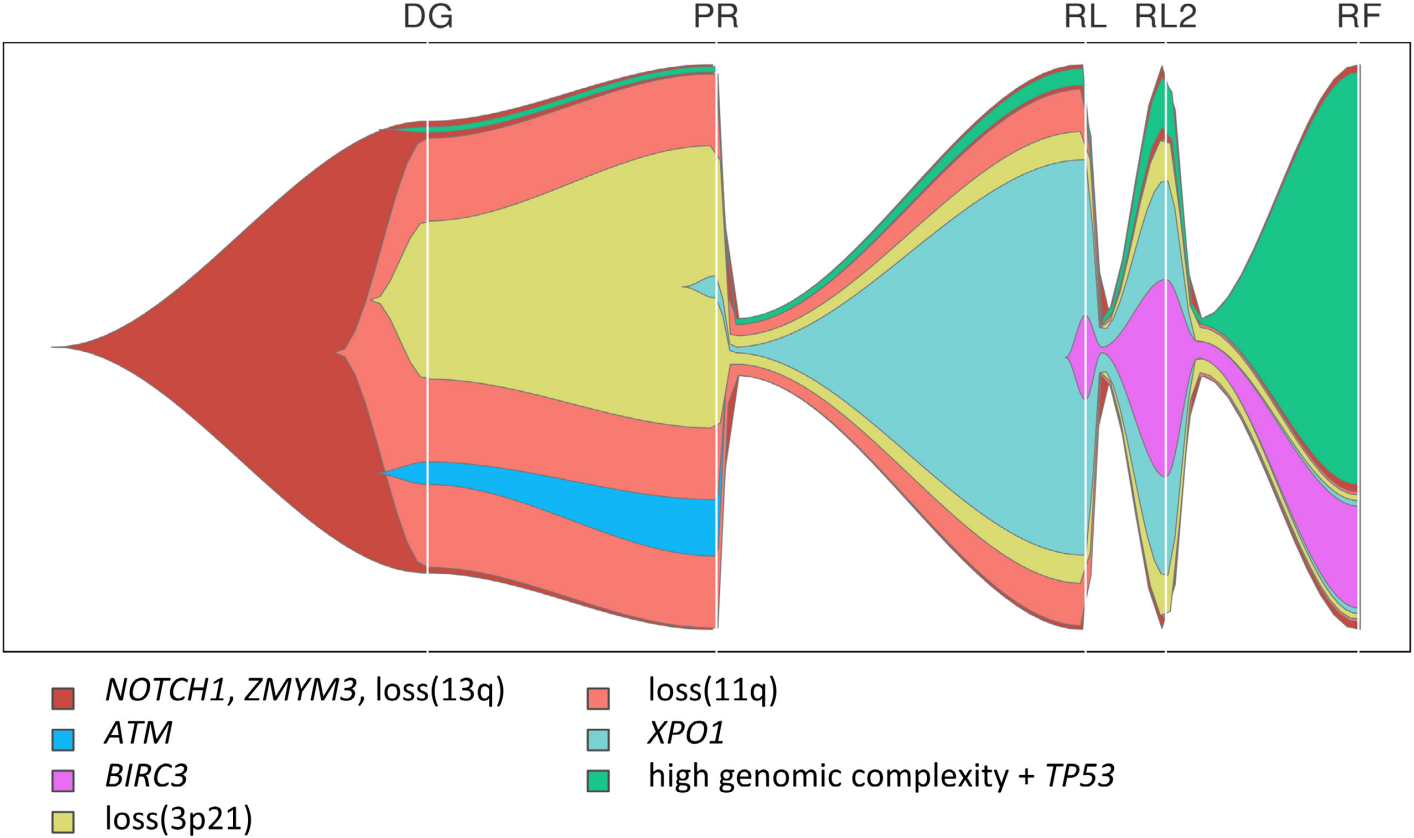
Reconstruction of leukemic cell evolution. Fish plot capturing estimated reconstruction of the mutational composition of cell populations and their evolution in time created by integrating bulk and single‐cell data. The emergence of a population within another is marked with an emergence of new colored stream and means that the given population contains new color‐coded change together with aberrations of population from which it originated. DG, diagnosis; PR, progression; RF, refractory disease phase; RL2, second relapse; RL, first relapse.

Most importantly, scRNA‐seq data combined with bulk analyses enabled us to reconstruct the development of leukemia cells during the course of the disease (Fig. 6). *NOTCH1* and *ZMYM3* gene mutations and loss(13q) were consistently present throughout the disease progression, indicating they most likely arose as primary events contributing to carcinogenesis. In the refractory phase of the disease, two populations of cells were present, one with loss(11q) and loss(3p21) corresponding to expression cluster 4 and the other with high genomic complexity forming expression cluster 2. In addition, cluster 4 contained cells with mutations in *BIRC3* and *XPO1* genes, while *TP53* mutation was most likely present in cluster 2.

## 5 Conclusion

The presented case of a female patient diagnosed with CLL at a relatively early age highlights the clinical challenges associated with disease management and treatment resistance. In our study, we took advantage of various single‐cell and bulk molecular methods and reconstructed the clonal evolution of CLL cells over time. We characterized two populations of refractory cells in terms of genomic and expressional changes and tracked them back in time before therapeutic intervention. We believe that conducting similar studies in the future may contribute to the early identification of treatment‐resistant rare subpopulation and to the adaptation of therapeutic regimens accordingly. In conclusion, our results shed light on the complexity of CLL and the need for comprehensive genomic analyses to understand better the disease dynamics and mechanism of treatment resistance.

## Conflict of interest

The authors declare no conflict of interest.

## Author contributions

JK designed and conceived the study, collected the clinical data and led the data analysis. TK prepared single‐cell sequencing libraries, analyzed the single‐cell data, interpreted the results, and wrote the manuscript. KR and BT supervised the single‐cell data analysis, library preparation and sequencing. JPP and VN processed and evaluated the LYNX data. SPa and JM processed and evaluated the *TP53* sequencing data. KP processed and evaluated the genomic microarray data. PJ and VB supervised single‐cell part of the project. MD and SPo supervised clinical part of the project. All authors contributed to the manuscript review.

### Peer review

The peer review history for this article is available at https://www.webofscience.com/api/gateway/wos/peer‐review/10.1002/1878‐0261.13663.

## Supporting information


**Fig. S1.** Visual evaluation of the batch effect presence in merged data using cells from progression (PR) processed in parallel in two separate experiments (PR_exp1 & PR_exp2).
**Fig. S2.** Graphical representation of copy number aberration estimation from single‐cell RNA sequencing data using InferCNV tool.
**Table S1.** Results of differential gene expression analysis comparing RF to other time points.
**Table S2.** Results of differential gene expression analysis comparing RL to previous time points (DG, PR).
**Table S3.** Summarized detected CNAs with their percentage representation at individual time points obtained using genomic arrays.

## Data Availability

All data in our study are available upon request.

## References

[1] Döhner H , Stilgenbauer S , Benner A , Leupolt E , Kröber A , Bullinger L , et al. Genomic aberrations and survival in chronic lymphocytic leukemia. N Engl J Med. 2000;343(26):1910–1916. 11136261 10.1056/NEJM200012283432602

[2] Hamblin TJ , Davis Z , Gardiner A , Oscier DG , Stevenson FK . Unmutated Ig V(H) genes are associated with a more aggressive form of chronic lymphocytic leukemia. Blood. 1999;94(6):1848–1854. 10477713

[3] Parikh SA , Strati P , Tsang M , West CP , Shanafelt TD . Should IGHV status and FISH testing be performed in all CLL patients at diagnosis? A systematic review and meta‐analysis. Blood. 2016;127(14):1752–1760. 26841802 10.1182/blood-2015-10-620864

[4] Mansouri L , Thorvaldsdottir B , Sutton L‐A , Karakatsoulis G , Meggendorfer M , Parker H , et al. Different prognostic impact of recurrent gene mutations in chronic lymphocytic leukemia depending on IGHV gene somatic hypermutation status: a study by ERIC in HARMONY. Leukemia. 2023;37(2):339–347. 36566271 10.1038/s41375-022-01802-y PMC9898037

[5] Puente XS , Beà S , Valdés‐Mas R , Villamor N , Gutiérrez‐Abril J , Martín‐Subero JI , et al. Non‐coding recurrent mutations in chronic lymphocytic leukaemia. Nature. 2015;526(7574):519–524. 26200345 10.1038/nature14666

[6] Landau DA , Tausch E , Taylor‐Weiner AN , Stewart C , Reiter JG , Bahlo J , et al. Mutations driving CLL and their evolution in progression and relapse. Nature. 2015;526(7574):525–530. 26466571 10.1038/nature15395 PMC4815041

[7] Nadeu F , Delgado J , Royo C , Baumann T , Stankovic T , Pinyol M , et al. Clinical impact of clonal and subclonal TP53, SF3B1, BIRC3, NOTCH1, and ATM mutations in chronic lymphocytic leukemia. Blood. 2016;127(17):2122–2130. 26837699 10.1182/blood-2015-07-659144 PMC4912011

[8] Hallek M , Cheson BD , Catovsky D , Caligaris‐Cappio F , Dighiero G , Döhner H , et al. iwCLL guidelines for diagnosis, indications for treatment, response assessment, and supportive management of CLL. Blood. 2018;131(25):2745–2760. 29540348 10.1182/blood-2017-09-806398

[9] Eichhorst B , Robak T , Montserrat E , Ghia P , Niemann CU , Kater AP , et al. Chronic lymphocytic leukaemia: ESMO clinical practice guidelines for diagnosis, treatment and follow‐up. Ann Oncol. 2021;32(1):23–33. 33091559 10.1016/j.annonc.2020.09.019

[10] Ding L , Ley TJ , Larson DE , Miller CA , Koboldt DC , Welch JS , et al. Clonal evolution in relapsed acute myeloid leukaemia revealed by whole‐genome sequencing. Nature. 2012;481(7382):506–510. 22237025 10.1038/nature10738 PMC3267864

[11] Malcikova J , Pavlova S , Kunt Vonkova B , Radova L , Plevova K , Kotaskova J , et al. Low‐burden TP53 mutations in CLL: clinical impact and clonal evolution within the context of different treatment options. Blood. 2021;138(25):2670–2685. 33945616 10.1182/blood.2020009530 PMC8703362

[12] Hallek M , Cheson BD , Catovsky D , Caligaris‐Cappio F , Dighiero G , Döhner H , et al. Guidelines for the diagnosis and treatment of chronic lymphocytic leukemia: a report from the international workshop on chronic lymphocytic leukemia updating the National Cancer Institute‐working group 1996 guidelines. Blood. 2008;111(12):5446–5456. 18216293 10.1182/blood-2007-06-093906 PMC2972576

[13] Hao Y , Hao S , Andersen‐Nissen E , Mauck WM , Zheng S , Butler A , et al. Integrated analysis of multimodal single‐cell data. Cell. 2021;184(13):3573–3587.e29. 34062119 10.1016/j.cell.2021.04.048 PMC8238499

[14] Finak G , McDavid A , Yajima M , Deng J , Gersuk V , Shalek AK , et al. MAST: a flexible statistical framework for assessing transcriptional changes and characterizing heterogeneity in single‐cell RNA sequencing data. Genome Biol. 2015;16:278. 26653891 10.1186/s13059-015-0844-5 PMC4676162

[15] Navrkalova V , Plevova K , Hynst J , Pal K , Mareckova A , Reigl T , et al. LYmphoid NeXt‐generation sequencing (LYNX) panel: a comprehensive capture‐based sequencing tool for the analysis of prognostic and predictive markers in lymphoid malignancies. J Mol Diagn. 2021;23(8):959–974. 34082072 10.1016/j.jmoldx.2021.05.007

[16] Miller CA , McMichael J , Dang HX , Maher CA , Ding L , Ley TJ , et al. Visualizing tumor evolution with the fishplot package for R. BMC Genomics. 2016;17(1):880. 27821060 10.1186/s12864-016-3195-z PMC5100182

[17] Malcikova J , Stano‐Kozubik K , Tichy B , Kantorova B , Pavlova S , Tom N , et al. Detailed analysis of therapy‐driven clonal evolution of TP53 mutations in chronic lymphocytic leukemia. Leukemia. 2015;29(4):877–885. 25287991 10.1038/leu.2014.297 PMC4396398

[18] Kubesova B , Pavlova S , Malcikova J , Kabathova J , Radova L , Tom N , et al. Low‐burden TP53 mutations in chronic phase of myeloproliferative neoplasms: association with age, hydroxyurea administration, disease type and JAK2 mutational status. Leukemia. 2018;32(2):450–461. 28744014 10.1038/leu.2017.230 PMC5808067

[19] Pavlova S , Smardova J , Tom N , Trbusek M . Detection and functional analysis of TP53 mutations in CLL. Methods Mol Biol. 2019;1881:63–81. 30350198 10.1007/978-1-4939-8876-1_6

[20] Ishioka C , Frebourg T , Yan YX , Vidal M , Friend SH , Schmidt S , et al. Screening patients for heterozygous p53 mutations using a functional assay in yeast. Nat Genet. 1993;5(2):124–129. 8252037 10.1038/ng1093-124

[21] Cobaleda C , Jochum W , Busslinger M . Conversion of mature B cells into T cells by dedifferentiation to uncommitted progenitors. Nature. 2007;449(7161):473–477. 17851532 10.1038/nature06159

