# Supplementary figures and images for: Unveiling the dynamics and molecular landscape of a rare chronic lymphocytic leukemia subpopulation driving refractoriness: insights from single‐cell RNA sequencing
Source: Mol Oncol. 2024 May 21;18(10):2541–53. doi: 10.1002/1878-0261.13663 (PMC11459043; doi:10.1002/1878-0261.13663)

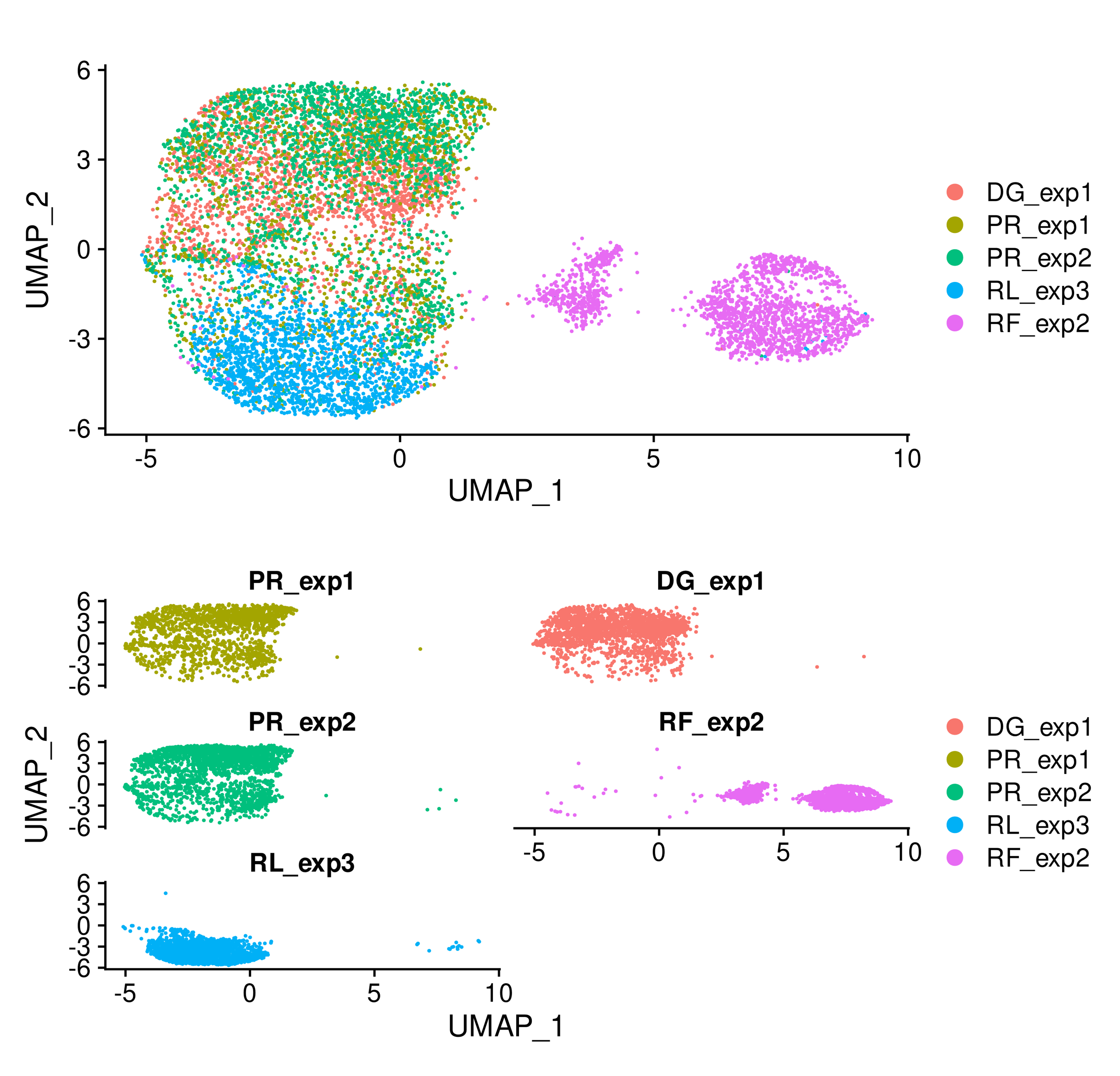

Supplement: Supplementary file 1 — Fig. S1. Visual evaluation of the batch effect presence in merged data using cells from progression (PR) processed in parallel in two separate experiments (PR_exp1 & PR_exp2). Fig. S2. Graphical representation of copy number aberration estimation from single‐cell RNA sequencing data using InferCNV tool. Table S1. Results of differential gene expression analysis comparing RF to other time points. Table S2. Results of differential gene expression analysis comparing RL to previous time points (DG, PR). Table S3. Summarized detected CNAs with their percentage representation at individual time points obtained using genomic arrays. [file MOL2-18-2541-s001.zip › mol213663-sup-0001-Suppl. fig1.tif]

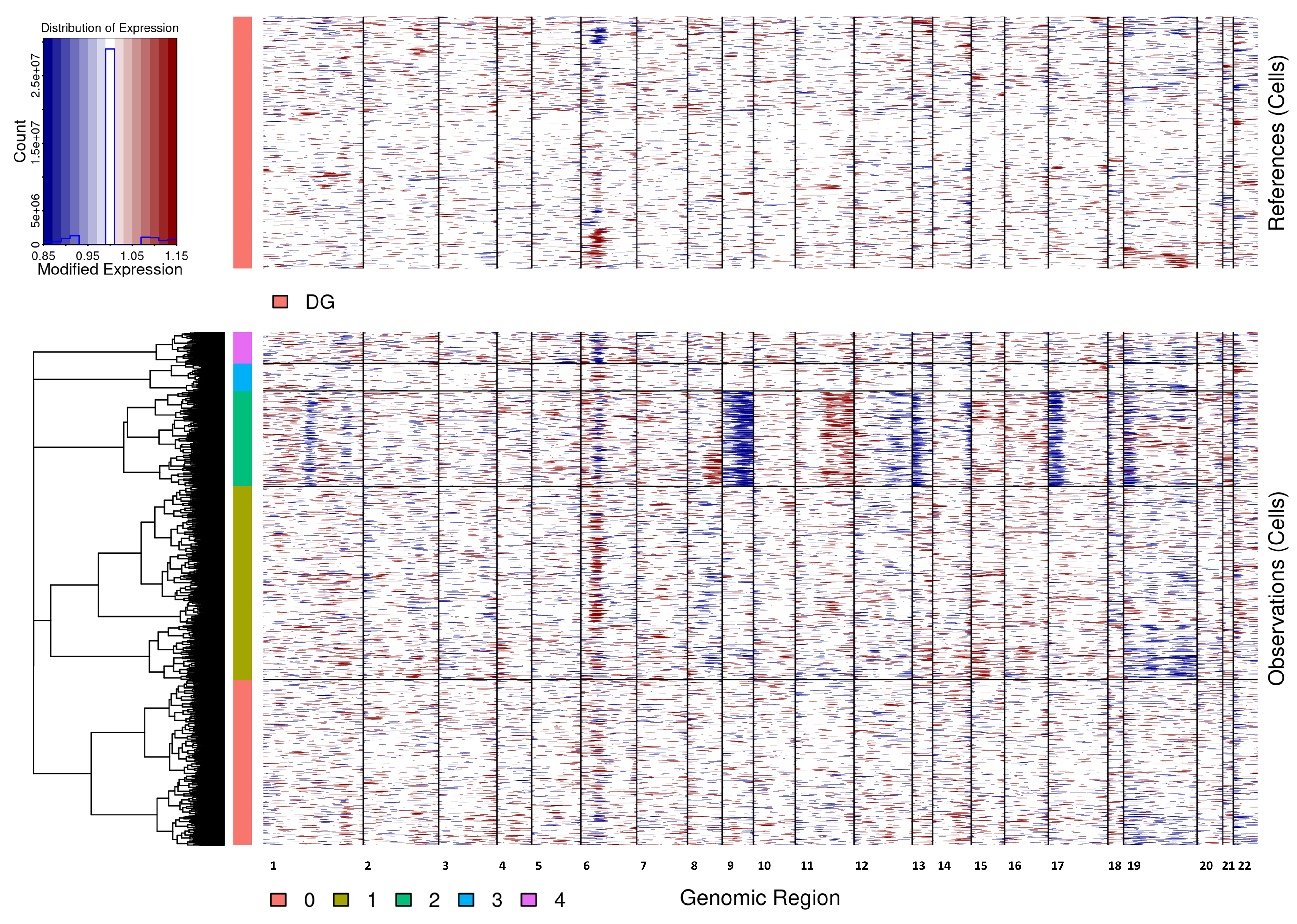

Supplement: Supplementary file 1 — Fig. S1. Visual evaluation of the batch effect presence in merged data using cells from progression (PR) processed in parallel in two separate experiments (PR_exp1 & PR_exp2). Fig. S2. Graphical representation of copy number aberration estimation from single‐cell RNA sequencing data using InferCNV tool. Table S1. Results of differential gene expression analysis comparing RF to other time points. Table S2. Results of differential gene expression analysis comparing RL to previous time points (DG, PR). Table S3. Summarized detected CNAs with their percentage representation at individual time points obtained using genomic arrays. [file MOL2-18-2541-s001.zip › mol213663-sup-0002-Suppl.fig2.tif]
